# 2529: Clinical determinants of clopidogrel responsiveness in a heterogeneous cohort of Caribbean Hispanics

**DOI:** 10.1017/cts.2017.239

**Published:** 2018-05-10

**Authors:** Dagmar Fredy Hernandez Suarez, Kyle Melin, Angel Lopez-Candales, Jorge Duconge

**Affiliations:** University of Puerto Rico School of Medicine, San Juan, Puerto Rico

## Abstract

OBJECTIVES/SPECIFIC AIMS: To determine the association between clinical characteristics and platelet reactivity in Hispanic patients on clopidogrel therapy. METHODS/STUDY POPULATION: A cross-sectional pilot study was performed in 58 Puerto Rican patients diagnosed with any type of vascular disease and actively receiving a maintenance dose of clopidogrel for at least 7 days. The study population was divided into 2 groups: Group I with non-high on-treatment platelet reactivity (TPR); Group II with high TPR. To determine the platelet function, P2Y12 reaction units (PRU) were obtained by VerifyNow^®^ P2Y12 assay (Accumetrics, USA). RESULTS/ANTICIPATED RESULTS: We studied a heterogeneous cohort of patients with coronary artery disease (57%), peripheral artery disease (30%), carotid artery stenosis (7%), cerebral artery aneurysm (3%), and stroke (3%) on clopidogrel therapy for secondary prevention of thromboembolic events. The mean TPR was 205±49 PRU (range: 61–304), with a prevalence of 28% patients with high TPR (PRU≥230). No significant clinical differences were found between the non-high TPR and high-TPR groups (*p*>0.05). However, multivariable logistic regression analysis showed that both diabetes mellitus (OR=7.5; CI: 1.01–51.9) and proton-pump inhibitors (OR=13.6; CI: 1.3–142.0) were independently correlated with high TPR (*p*<0.05) after adjusting for other clinical variables. DISCUSSION/SIGNIFICANCE OF IMPACT: These results provide new insight into the importance of clinical characteristics on platelet reactivity in this Caribbean population. Further studies are warranted to determine whether important clopidogrel pharmacogenes are related with platelet function in Hispanics, as well as the role of TPR in guiding antiplatelet therapy and predicting future adverse cardiovascular events in this population.

